# Factors associated with acceptability of child circumcision in Botswana -- a cross sectional survey

**DOI:** 10.1186/s12889-016-3722-5

**Published:** 2016-10-06

**Authors:** Mpho Keetile, Motsholathebe Bowelo

**Affiliations:** Department of Population Studies, University of Botswana, Private Bag UB 00705, Gaborone, Botswana

**Keywords:** Acceptability, Child, Safe Male circumcision, Factors, Botswana

## Abstract

**Background:**

Safe male child circumcision has been recently adopted as a potential strategy to prevent HIV/AIDS transmission in later life in Botswana.

**Methods:**

Data used was derived from a cross-sectional survey, the Botswana AIDS Impact Survey (BAIS) IV, conducted in 2013. A total sample of 7984 respondents in ages 15–64 years who had successfully completed the individual questionnaire during the survey were selected and included for analysis. Both descriptive and multivariable analyses were used to explore factors associated with acceptability of child circumcision. Data was analysed using SPSS version 22 program.

**Results:**

Results indicate that about 84 % of participants said they would circumcise their male children aged 18 years and below, while 93 % were aware of the safe male circumcision program. Bivariate analyses results show that acceptability of child circumcision was significantly associated with sex, age, education, religion, residence, HIV status of the parent, fathers circumcision status, father's intention to circumcise and parent's knowledge about the safe male circumcision program. Multivariable analyses results indicate positive association between respondent's HIV positive status (OR, 3.5), Men's circumcision status (OR, 3.7), men's intention to circumcise (OR, 9.3) and acceptability of child circumcision.

**Conclusion:**

Results of this study indicate some relatively high acceptability levels for child circumcision. Some individual behavioural factors influencing acceptability of child circumcision were also identified. This study provides a proper understanding of factors associated with acceptability of child circumcision which will ultimately enhance the successful roll-out of the school going children circumcision program in Botswana.

## Background

Male circumcision is one of the oldest surgical procedures, traditionally accepted as a mark of cultural identity or religious importance, or for perceived health benefits such as improved penile hygiene or reduced risk of infection [1]. Traditionally, circumcising societies and religious sects have used the procedure for cultural and religious purposes [2]. Recent epidemiological evidence has shown that safe circumcision reduces the risk of acquiring HIV infection in heterosexual males by 50–60 % [2]. It is on the basis of this evidence that several African countries with high prevalence of HIV are now expanding access to safe circumcision [3, 4]. Observational studies suggest that the protective effect of male circumcision is similar if circumcision occurs early in life [5]. The immediate focus of circumcision for HIV prevention has been on adolescents and adult men, but a longer-term HIV prevention strategy has to include the provision of child circumcision services.

Child circumcision is routinely practised in most countries in the Middle East (in countries such as, Egypt, the Islamic Republic of Iran, Jordan, Lebanon, the Syrian Arab Republic, Turkey and Yemen), Israel [6–8], the USA [9] and some West African countries, including Senegal, Ghana and parts of Nigeria [10, 11]. This type of circumcision is done mainly for religious and cultural purposes. Studies have provided that the best age to perform circumcision is in childhood and it has been shown to have a better protective effect than those performed at any other age [12]. It is also safer, easier and less costly but it is not widespread in southern Africa countries including Botswana [13].

Based on studies conducted in some parts of sub Saharan Africa [SSA] in particular the randomized clinical trials (RCTs) conducted in Uganda [2]; Kenya [3] and South Africa [4], safe male circumcision has a protective effect against HIV as well as reducing incidences of other sexually transmitted infections (STIs) like genital ulcers, human papilloma virus (HPV) and Chlamydia in female partners. Meanwhile child circumcision is recognised as a long term preventive strategy to reduce new infections particularly in later life as recommended by UNICEF and WHO [1]. In 2009, the Ministry of Health (Botswana) launched the safe male circumcision (SMC) policy as part of the comprehensive strategy on HIV prevention. Following this decision, circumcision services are being extended many public health facilities free of charge with the intention to increase accessibility of the service to as many males as possible. As a strategy to enhance uptake of circumcision services, there has been some efforts to reach out to school going children. The benefits of child circumcision compared to adult circumcision have been emphasized in several studies. For instance, in a qualitative study conducted in Zimbabwe, it was observed that circumcising children protected them against HIV transmission in later life [4, 7, 14, 15].

Previous studies have shown high adult male circumcision acceptability rate in Botswana and in the region [14, 16, 17]. Kebaabetswe et al. [18], suggested that circumcision for the children of Botswana would be highly acceptable, and believed that parents in Botswana—as in most developed countries worldwide be offered the option of hospital based circumcision for their male children to protect them from the acquisition of HIV. Generally, male child circumcision is not yet performed in most southern African countries and there are questions regarding its acceptability, feasibility, safety and optimal approaches to widespread implementation [13].

Although Botswana has adopted safe male circumcision as a key strategy against HIV/AIDS transmission, there is little information on factors influencing parental acceptance of child circumcision. Botswana has been running SMC program since 2009 and has not yet met its target of circumcising 80 % of males by 2016 [13]. Recently (in 2015), the government introduced Early Infant Safe Male Circumcision (EISMC) program as an add-on to a series of response programs to reduce HIV transmission in later life. The social and behavioural context of the countries implementing male circumcision programs might provide the reasons why set targets for SMC are not being met. Very little research has been carried out to explore factors (especially behavioural) influencing willingness to accept child male circumcision in the general population of Botswana. Plank et al. [13] conducted a study in South Eastern part of Botswana among women to assess whether they would accept their new-born male children to be circumcised. Although their study provided vital insights about acceptability of infant circumcision, the main limitations of the study was that it did not include men who are also role players in reproductive decisions of their families and was limited in scope and coverage.

This study uses nationally representative data and also includes men who are vital in decision making of the reproductive health of their families. The main aim of this study was to assess factors associated with acceptability of safe male child circumcision in Botswana. The study of this nature is essential in the context of Botswana where HIV prevalence rate continues to be high, and the SMC program has failed to reach the expected target. The study will serve to guide successful rollout of different SMC programs in Botswana. Moreover, since child circumcision has been found to decrease the risk of HIV infection among men in later life, it is important to determine its acceptability in the general population as a potential HIV prevention strategy.

### Theoretical framework

The study employed Theory of Reasoned Action, [TRA] developed and several times modified by Ajzen and Fishbein [19–21]. TRA proposes that behavioural intentions are a combined function of the attitude toward performing a particular behaviour in a given situation and of the norms perceived to govern that behaviour multiplied by the motivation to comply with those norms [19]. This theory assumes that human beings are usually quite rational and make systematic use of the information available to them. People consider the implications of their actions before they decide to engage or not engage in a given behaviour [21].

As child circumcision is recommended for medical reasons [especially prevention of HIV acquisition in later life], mothers and fathers who may choose circumcision must also believe that circumcising their children may reduce chances of HIV acquisition later in life. The study attempted to determine factors influencing parent’s decision to circumcise their children. We chose this model mainly because, we believe that constructs of this model are key in informing parental decision on accepting child circumcision.

**Fig. 1:**
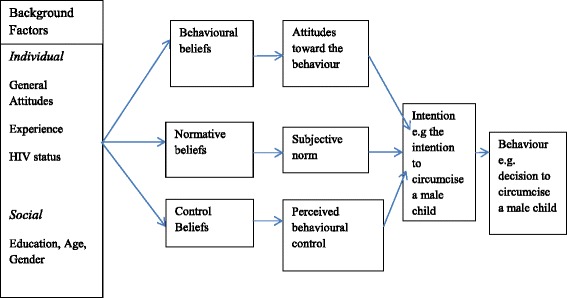
Theory of reason action and planned behaviour. Adapted and modified from Brenda Wells Dyal, 2006

The assumption of TRA is that most behaviours of social relevance are under volitional control and that a person’s intention to perform or not perform behaviour is the immediate determinant of that action [21]. A person’s intention regarding routine circumcision is determined by personal and social influences. One personal factor is the person’s evaluation of the outcome of circumcision, which can be either positive or negative. Parents who believe circumcision is necessary for reduction of HIV transmission may choose the procedure. Meanwhile parents who believe otherwise may have negative evaluation of circumcision and may choose not to circumcise their children. Subjective norm is the other determinant of a person’s intention which is a person’s perception of the social pressures applied to perform the behaviour [21]. As illustrated in Fig. 1, an individual’s intentions and behaviours are influenced by certain background factors which include individual, social and information factors.

The above figure shows one way in which the intentions and behaviour can be represented. There are beliefs which are assumed to influence attitudes, subjective norms, and perceived behavioural control which, in turn, produce intentions and behaviour [21]. Feng and Wu [22], also state that, intention is the best predictor of behaviour, and it is a function of the person’s attitude towards performing the behaviour and general subjective norms concerning the performance of that behaviour. For example, if a father intends to circumcise his child in future, he may eventually do so or he may also choose not to circumcise his child given the prevailing circumstances at the time. The Theory of Reasoned Action states that beliefs determine attitudes and subjective norms which then determine intention and the corresponding behaviour [20]. For instance, if the child’s father is circumcised, the father may also believe circumcision to be normal or necessary for their child. In addition, if most males in the community or society have been circumcised, the parents, in particular the father can subjectively intend to circumcise their children or decide otherwise. Although constructs of the TRA discussed above not been precisely used in the paper, notions of the TRA have been used to understand why would parents accept or reject child circumcision.

## Methods

This study used data derived from the 2013 Botswana AIDS Impact Survey (BAIS-IV), which is the fourth and latest of a series of nationally representative demographic surveys aimed at providing up to date information on the HIV /AIDS epidemic. The objectives of the BAIS IV included providing latest information on the national HIV prevalence and incidence estimates among the population 18 months and above; to provide indicative trends in sexual and preventive behavior among the population aged 10–64 years; and provide a comparison between HIV rate, behavior, knowledge, attitude, poverty and cultural factors that are associated with the pandemic with estimates derived from previous surveys.

All districts and major urban centres became their own strata. Enumeration Areas [EAs] were grouped according to ecological zones in rural districts and according to income categories in cities/towns. Geographical stratification along ecological zones and income categories was undertaken to improve the accuracy of the survey data because of the homogeneity of the variables within each stratum.

BAIS-IV employed a national two stage sample survey design. The first stage was the selection of EAs as Primary Sampling Units [PSUs] selected with probability proportional to measures of size (PPS), where measures of size [MOS] were the number of households in the EA as defined by the 2011 Population and Housing Census. EAs were selected with probability proportional to size. In the second stage of sampling, the households were systematically selected from a fresh list of occupied households prepared at the beginning of the survey's fieldwork [i.e. listing of households for the selected EAs] and households were drawn systematically. Estimates for response rates showed that 83.9 % of persons aged 10–64 years answered individual questions.

Data collection for BAIS IV was done by trained enumerators and was led by the National AIDS Coordinating Agency. Data collection was done using smart phone tablets instead of the conventional paper based method. Twenty-nine teams comprising of 6enumerators, and 1 supervisor per team collected data as well as testing the sampled population for HIV. Each team was expected to cover between 10 and 11 enumeration areas during the survey and there were 301 enumeration areas covered. This study was commissioned by the government of Botswana, and was implemented by National AIDS Coordinating Agency under the Office of the President. Ethical clearance was granted by the Ministry of Health’s Health Research and Development Division and all ethical issues were considered.

Sampled population for BAIS IV was 9807 and responded population was 8231 [10–64 years], yielding a response rate of 83.9 %^1^. Sample selection for this paper was such that a total of 7984 individuals who had successfully completed the individual questionnaire, and were aged 15–64 years were considered for analysis. The final selection of the sample yielded 3744 males and a total of 4240 females [total sample = 7984], using SPSS data selection command.

### Dependent variable

The dependent variable used in this study is acceptability of child circumcision, measured by the following question; *“Suppose you had male children aged below 18 years would you get them circumcised”.* There were three outcomes for this question, yes = 1, no = 2 and unsure = 3. The final outcomes for this paper are two, yes = 1 [acceptance] and no = 0 [refusal]. 69 respondents who reported that they were unsure were filtered out.

### Independent variables

This study investigates the effects of the following variables on the respondent’s decision to accept child circumcision;

i) Knowledge about safe male circumcision-This variable was derived from the following survey question; “Have you ever heard of Safe Male Circumcision or SMC program? Possible responses were yes = 1 and no = 2.
ii) Religion of respondent^2^; This was derived from a question asking respondents about their main religious affiliations. The following religions were listed; Christianity = 1, Islam = 2,Bahai = 3, Hinduism = 4,Badimo = 5,No religion = 6 and other religions (open responses). The final variable codes were as follows; Christianity = 1, Islam, Bahai, Hinduism, Badimo and other religions were grouped together and coded as Other non-Christian religions = 2 and no religion was coded 3.
iii) Men’s circumcision status-Men’s circumcision status was derived from the survey question; “Are you circumcised”? Yes = 1 & no = 2, don’t know response was filtered out.
iv) Intention to be circumcised; This was derived from a question asking uncircumcised men about their intension to circumcise in the next 12 months: “Do you intend to get circumcised in the next 12 months. Responses were yes = 1 & No = 2
v) HIV status of respondent: This was derived from the question: “what was the result”? Positive = 1, Negative =2, Don’t want to tell = 3 and Don’t know = 4. This was a follow-up question to the question asking respondents whether they were told/given results for their last HIV test. The don’t want to tell and don’t know responses were treated as missing values and were filtered out and not included in the analysis. Control variables used are age, education, marital status, and place of residence.

Data analyses implored in this paper include both bivariate and multivariable analyses. BAIS IV was a national study and inference is made from the sample to the entire population. Bivariate analysis results are presented as percentages and are used to present the association between acceptability of child circumcision, behavioural and control variables. For multivariable analyses logistic regression is used to identify key factors associated with acceptability of child circumcision. Logistic regression results are presented in the form of unadjusted [Model I-gross effects] and adjusted odds ratios [Model II-net effects], together with their 95 % confidence intervals [C.I.]. Logistic regression results explain the probability of accepting child circumcision in a particular category of variable in comparison with the reference category, while controlling other factors. Data analysis was done using Statistical Package for Social Sciences version 22 program [SPSS].

### Logit Model I

Model I presents the probability of accepting child circumcision based on socio-demographic factors [e.g. sex, age, religion, education, marital status and place of residence]. The regression equation for model I take the form;

$$ \begin{array}{l}\kern3.50em p/\\ {}1n\ \left(1 - p\right) = \beta 0 + \beta 1\ X\end{array} $$

Where *p* is the probability that the respondent is likely to accept their male child to be circumcised. *1-p* is the probability that the respondent will not accept their male child to be circumcised. *β0 and β1X* are components of the regression equation, the βs represent regression coefficients and *Xs* represent a set of independent variables. The key independent variables used are respondent’s age, sex, residence, religion and education.

### Logit Model II

Model II measures the probability of accepting child circumcision based on a set of factors while controlling for potential confounders. Model II introduces behavioural factors [The following behavioural factors are included; HIV status of the respondent, men’s circumcision status, men’s intention to circumcise and knowledge about safe male circumcision] which may influence the respondent’s decision to circumcise their male children. The regression equation fitted to data takes the form;

$$ \begin{array}{l}\kern4em p/\\ {}1n\ \left(1 - p\right)=\beta 0 + \beta 1\ {X}_1{X}_2\dots \dots \dots {X}_k\end{array} $$

Where *p* is the probability that the respondent is likely to accept their male child to be circumcised. *1-p* is the probability that the respondent will not accept their male child to be circumcised. *β0* and *β1X* are components of the regression equation, the βs represent regression coefficients and *Xs* represent a set of independent variables and X_k_ is an array of behavioral independent variables which may influence the respondent’s decision to circumcise their male children. These are potential confounders on the decision to circumcise a male child.

### Ethical considerations

Ethics approval for Botswana AIDS Impact Survey IV was granted by the Health Research and Development Division in the Ministry of Health. During data collection for BAIS IV written informed consent and assent were sought from respondents who were informed about the purpose and design of the study, and assured that participation was voluntary and confidential.

## Results

### Sample description

Table 1 presents the sample population based on socio-demographic characteristics and a set of behavioural factors. Results indicate that there were slightly a high proportion of females (53 %) than males (47 %) in the sample. Respondents in ages 15–34 years accounted for 59 % of the sample, while those in ages 35–44 and 45–64 years both accounted for 20 %. Respondents with secondary education (57 %) were the prominent education group in the sample. Meanwhile respondents from rural areas accounted for 64 %. The predominant religious affiliation in the sample is Christianity (86 %) followed by no religious affiliation (9 %).

**Table 1:** Sample characteristics

Variable	Percent	Number
Sex		
Male	46.9	3744
Female	53.1	4240
Age		
15–24	29.8	2379
25–34	29.7	2371
35–44	20.3	1620
45–54	12.7	1013
55–64	7.5	601
Education		
Primary/less	21.1	1684
Secondary	56.9	4542
Tertiary/higher	22.0	1758
Place of residence		
Urban	35.8	2858
Rural	64.2	5126
Religion		
Christianity	86.3	6890
Other non-Christian	4.3	343
No religion	9.4	751
Suppose you had male children aged below 18 years would you get them circumcised?		
Yes	83.9	6698
No	16.1	1286
HIV status of respondent?		
Positive	19.0	1066
Negative	81.0	4547
Men’s circumcision status?		
Circumcised	25.4	950
Uncircumcised	74.6	2794
Men’s intention to circumcise?		
Yes	55	1536
No	45	1258
Knowledge about circumcision		
Yes	93.1	7433
No	7.9	551

When considering behavioural factors, 84 % of respondents said that they would accept their male children aged below 18 years to be circumcised. Meanwhile 19 % of respondents in the sample reported that they were HIV positive, while 25 % of men in the sample reported that they were circumcised. Furthermore, results also indicate that about 55 % of uncircumcised men in the sample had the intention to circumcise in the next 12 months, whereas 93 % of study participants knew about the safe male circumcision program.

### Acceptability of child circumcision

Table 2 show acceptability of child circumcision among respondents by sample characteristics. Results indicate that a slightly high proportion of females (88 %) than males (84 %) reported that they would get their male children to be circumcised. When considering age of participants, a relatively low proportion of respondents in ages 25–34 years (84 %) than in other ages (over 85 %) said they would accept their male children to be circumcised. Quite conversely, a significantly small proportion of respondents with tertiary education (83 %) than those with primary (92 %) and secondary education (88 %) reported that they would get their male children to be circumcised. Results also indicate that slightly more respondents in urban areas (88 %) than rural areas (85 %) reported that they would accept their male children to be circumcised.

**Table 2:** Acceptability of child circumcision among respondents by sample characteristics

Variable	Accept child to be circumcised		
	Yes	No	*n*
Sex			
Male	83.5	16.5	3744
Female	88.1	13.9	4240
	*Chi-square = 8.918*	*P = 0.003*	
Age			
15–24	85.7	14.3	2379
25–34	83.7	16.3	2371
35–44	88.2	11.8	1620
45–54	88.1	11.9	1013
55–64	85.9	14.1	601
	*Chi-square = 17.353*	*P = 0.002*	
Education			
Primary/less	92.1	8.9	1684
Secondary	88.2	11.8	4542
Tertiary/higher	83.1	16.9	1758
	*Chi-square = 24.426*	*P = 0.000*	
Place of residence			
Urban	88.1	10.9	2858
Rural	84.6	15.4	5126
	*Chi-square = 15.205*	*P = 0.000*	
Religion			
Christianity	86.4	13.6	6890
Other non-Christian	85.5	14.5	343
No religion	78.2	21.8	751
	*Chi-square = 34.716*	*P = 0.000*	
HIV status of respondent?			
Positive	90.1	9.9	1066
Negative	86.1	13.9	4547
	*Chi-square = 12.092*	*P = 0.001*	
Men’s circumcision Status?			
Circumcised	94.4	5.6	950
Uncircumcised	80.9	19.1	2794
	*Chi-square = 85.810*	*P = 0.000*	
Men’s intention to circumcise?			
Yes	92.2	8.8	1536
No	63.6	36.4	1258
	*Chi-square = 293.010*	*P = 0.000*	
Knowledge about safe male circumcision program			
Yes	87.6	12.4	7433
No	58.6	41.4	551
	*Chi-square = 294.201*	*P* = 0.000	

Furthermore, a significantly high proportion of Christian (86 %) and participants of other non-Christian religions (86 %) reported that they would circumcise their male children compared to individuals who said they do not affiliate to any religion (78 %). A higher proportion of respondents who reported that they were HIV positive (90 %) compared to those who reported that they were negative (86 %) said they would accept circumcision of their male children. A significant proportion of circumcised men (94 %) than uncircumcised men (81 %) reported that they would circumcise their children. On the other hand, 92 % of men who intended to be circumcised in the next 12 months, said they would accept circumcision of children, compared to only 64 % of men who said they did not intend to get circumcised themselves. Results also show that about, 88 % of respondents who knew about the safe male circumcision program, reported that they would circumcise their male children compared to those who did not know about the program (59 %).

### Logistic regression results for the probability of accepting child circumcision among respondents

#### Model I results

Table 3 shows the logistic regression results for the probability of accepting child circumcision among respondents. Results indicate that sex of the respondent is a significant factor for the probability of accepting child circumcision when considering demographic variables only. For instance, women were more (OR 1.18, C.I. =1.02-1.38) willing to accept their children to be circumcised compared to men. Young adults in ages 15–24 years and 25–34 years respectively, were less willing to accept their children to be circumcised compared to adults in ages 55–64 years. Education was not significantly associated with accepting child circumcision. When considering place of residence, respondents from rural areas were less willing (OR 0.76, C.I. = 0.65-0.89) to accept their children to be circumcised. Moreover, results indicate that the odds of accepting child circumcision were significantly higher among Christians (OR 1.55, C.I. = 1.22-1.97) and other non-Christian religions (OR 1.63, C.I. = 1.05-2.54) respondents than among individuals with no religion affiliation.

**Table 3:** Odds ratios (OR) and 95 % confidence Intervals for the probability of accepting child circumcision

Variables	Model I (Unadjusted ORs)		Model II (Adjusted ORs)	
	OR (95 % CI)	*p*-value	OR (95 % CI)	*p*-value
Socio-demographics				
Sex				
Male	1.00		1.00	
Female	1.18 (1.02–1.38)	0.023	0.83 (0.55–1.25)	0.998
Age				
15–24	0.66 (0.45–0.97)	0.033	0.72 (0.67–0.77)	0.000
25–34	0.64 (0.44–0.93)	0.020	1.34 (1.25–1.43)	0.000
35–44	0.99 (0.68–1.48)	0.998	1.41 (1.31–1.50)	0.000
45–54	1.33 (0.86–2.04)	0.198	1.31 (1.21–1.41)	0.000
55–64	1.00		1.00	
Education				
Primary/less	1.00		1.00	
Secondary	0.62 (0.19–2.03)	0.425	0.60 (0.17–2.01)	0.422
Tertiary/higher	0.97 (0.29–3.21)	0.961	0.93 (0.26–3.19)	0.951
Place of residence				
Urban	1.00		1.00	
Rural	0.76 (0.65–0.89)	0.001	0.96 (0.93–0.99)	0.027
Religion				
Christianity	1.55 (1.22–1.97)	0.000	2.36 (2.27–2.45)	0.000
Other non-Christian	1.63 (1.05–2.54)	0.030	3.10 (2.91–3.31	0.000
No religion	1.00		1.00	
Behavioural factors				
HIV status				
Negative			1.00	
Positive			3.51 (3.34–3.69)	0.000
Men’s circumcision status				
Circumcised			3.69 (3.58–3.81)	0.000
Uncircumcised			1.00	
Men’s Intention to circumcise?				
Yes			9.32 (9.02–9.64)	
No			1.00	
Knowledge safe male circumcision program about circumcision				
Yes			0.85 (0.79–0.89)	0.000
No			1.00	

#### Model II results

Model II introduces behavioural factors. Results indicate that when behavioural factors were introduced in the model, sex was not a significant predictor of accepting male child circumcision. The odds of willingness to accept child circumcision was high among respondents in ages 25–34 (OR 1.34, C.I. = 1.25-1.43), 35–44 (OR 1.41, C.I. = 1.31-1.50) and 45–54 years (OR 1.31, C.I. = 1.21-1.41). Respondents residing in rural areas were less willing (OR 0.96, C.I 0.93-0.99) to accept their male children to be circumcised compared to those in urban areas. Christians (OR 2.36, C.I. = 2.27-2.45) and respondents of other non-Christian religions (OR 3.10, C.I. = 2.91-3.31) were more willing to accept child circumcision than individuals with no religion.

Another observation is that individuals who reported that they were HIV positive were more willing to accept child circumcision than those who said they were HIV negative. The odds of willingness to accepting male child circumcision were significantly higher among men who were circumcised (OR 3.69, C.I. = 3.58-3.81) than those who were not circumcised. Men who had the intention to be circumcised in the next 12 months after the survey were nine times more willing (OR 9.32, C.I. = 9.02-9.64) to accept circumcision of their male children compared to those who did not have any intention to circumcise in the next 12 months. Quite conversely, respondents who said they knew about safe male circumcision program were less willing to circumcise (OR 0.85, C.I. = 0.79-0.89) their children than those who did not know about the program.

## Discussion

The government of Botswana has been running a series of response programs aimed at reducing HIV transmission. However, since the introduction of the SMC program in 2009, it has not yet been able to meet its target of circumcision of 80 % of males by 2016. Meanwhile, the results of this study indicate a relatively high level of acceptability (84 %) of child circumcision in the general population of Botswana. Some previous studies in SSA region focusing on the general male population have also shown high levels of acceptability of circumcision [13, 14, 16, 17]. In order to increase scope and coverage of circumcision services, the government of Botswana has adopted child circumcision in the HIV/AIDS prevention package. This is done, in a context where little is known about the acceptability of child circumcision in the general population. These results provide impetus for the successful roll out of child circumcision services, especially among the school going children.

Acceptability of child circumcision in Botswana is associated with gender of respondents, with women more willing to accept their male children to be circumcised. On the other hand, some studies in SSA have shown that men have more decision-making power to decide over child circumcision than do mothers [23–25]. These studies have shown that when parents disagreed about circumcising their male children, men’s decision not to circumcise tended to predominate, regardless of whether the mother favored circumcision. Even qualitative studies have shown similar findings that men’s decision to accept child circumcision is instrumental. For instance a qualitative study in Zimbabwe has shown that both male and female participants concurred that men have the ultimate decision to circumcise their children [15]. Child circumcision programs should include education, information and communication materials for men to enhance acceptability.

When controlling for age, we found that men’s intention to circumcise was also significantly associated with the likelihood of accepting child circumcision. The theory of reasoned action posits that intention is the best predictor of behaviour, and it is the function of person’s attitude towards performing behaviour or taking action towards behaviour. For instance, a man who validates circumcision may have the intention to be circumcised and that intention may trickle down to the desire to have their male child circumcised. This is so because a man who is willing to be circumcised has the belief that circumcision is acceptable, hence positive attitude and subjective norms which then determine and reinforce the intension to accept child circumcision.

The findings of this study also show that circumcised men were more willing to accept their male children to be circumcised. Another study in Zambia also found that father’s circumcision status was one of the reasons for their positive decision to accept circumcision of their children [26]. Furthermore, in Nyanza province, Kenya parents also identified father’s circumcision status as being one of the strongly associated factors with decision favouring child circumcision [27]. A father’s intention regarding accepting child circumcision is determined by personal and social influences. A circumcised man perceives circumcision as a socially acceptable practice hence they would easily accept it. A personal evaluation of the outcome of the procedure will have a direct influence on their decision to circumcise their male children.

Meanwhile respondents who reported that they were HIV positive were more willing to accept child circumcision than those who said they were HIV negative. This is the expected norm, that HIV positive parents would be eager to circumcise their male children. This finding is in conformity with what was found in Kampala, Uganda where HIV positive parents showed high propensity and willingness to circumcise their children than HIV negative parents [28]. There is need to understand the complications underlying the attitude of HIV negative parents to refuse circumcision of their male children.

Religion was found to be one of the factors influencing acceptability of child circumcision. Christians and other non-Christian religions such as Muslim, Hindu were more willing to accept their male children to be circumcised than those who reported to be non-religious. These findings are consistent with results from other studies such as in Malawi [29], where aacceptability was higher in central and southern districts where MC is practiced by a minority Muslim group (Yao) while in Kenya church membership is associated with being circumcised [30]. In Zambia, there is prevalent perception that circumcision is linked with Islam while Christians believe that they should practice circumcision since Jesus was circumcised and the Bible teaches the practice [31].

### Limitations

Strengths of our study included a relatively large sample size and a diverse population of respondents. However, the use of secondary data limited the scope of this study to variables within the dataset. Like most demographic surveys, the absence of qualitative data denies this analysis an in-depth understanding and explanation of patterns observed in the quantitative analysis. For instance, results indicate that HIV negative respondents were less likely to accept their male children to be circumcised and only qualitative results would explain this observed pattern. Despite these limitations, the data provides important insights into the potential of child safe male circumcision in Botswana as a strategy to mitigate HIV infection in later life.

## Conclusion

This study identified some individual behavioural factors influencing decisions of people to accept or reject child circumcision. For instance, willingness to circumcise male child was observed to be positively associated with men’s circumcision status, men’s intention to circumcise, and respondent’s HIV status. The findings of the study provide evidence base for the successful implementation and rolling-out of the broad safe male circumcision program targeting school going children and the recently introduced EISMC program. There is need to improve demand creation strategies for the EISMC program in order to achieve far-reaching acceptance levels.

## Abbreviations

BAIS: Botswana AIDS Impact Survey
EISMC: Early infant safe male circumcision
HIV: Human immune virus
RCTs: Randomised clinical trials
SMC: Safe male circumcision
TRA: Theory of Reason Action

## Footnotes

1 Refer to BAIS IV report online for further information- http://www.cso.gov.bw/images/aids_summary.pdf

2 Religion has been identified as one of the key determinants of circumcision
